# The association between stair climbing and modifiable cardiovascular disease risk factors: the Suita Study

**DOI:** 10.1265/ehpm.23-00323

**Published:** 2024-05-03

**Authors:** Ahmed Arafa, Yuka Yasui, Yuka Kato, Chisa Matsumoto, Yoshihiro Kokubo

**Affiliations:** 1 Department of Preventive Cardiology, National Cerebral and Cardiovascular Center, Suita, Japan; 2 Department of Public Health, Faculty of Medicine, Beni-Suef University, Beni-Suef, Egypt; 3 Graduate School of Human Life and Science, Doshisha Women’s College of Liberal Arts, Kyoto, Japan; 4 Division of Health Sciences, Osaka University Graduate School of Medicine, Suita, Japan; 5 Department of Cardiology, Center for Health Surveillance and Preventive Medicine, Tokyo Medical University Hospital, Shinjuku, Japan

**Keywords:** Stair climbing, Physical activity, Cardiovascular disease, Obesity, Risk factors

## Abstract

**Background:**

Stair climbing is a readily available form of physical activity with potential cardiovascular benefits. This study aimed to investigate the association between stair climbing and numerous modifiable cardiovascular disease (CVD) risk factors.

**Methods:**

In this cross-sectional study, we used data from 7282 Japanese people (30–84 years) residing in Suita City, Osaka. CVD risk factors and stair climbing frequency were assessed during the Suita Study health examination. Logistic regressions were used to calculate the odds ratios (ORs) and 95% confidence intervals (95% CIs) for CVD risk factors across stair climbing frequencies.

**Results:**

After adjustment for age, sex, lifestyle, and medical conditions, stair climbing >60% of the time, compared to <20% of the time, was inversely associated with obesity, smoking, physical inactivity, and stress: ORs (95% CIs) = 0.63 (0.53, 0.75), 0.81 (0.69, 0.96), 0.48 (0.41, 0.55), and 0.67 (0.58, 0.78), respectively (p-trends < 0.05).

**Conclusion:**

Stair climbing was inversely associated with obesity, smoking, physical inactivity, and stress; suggesting a potential role for cardiovascular disease prevention.

## Graphical Abstract

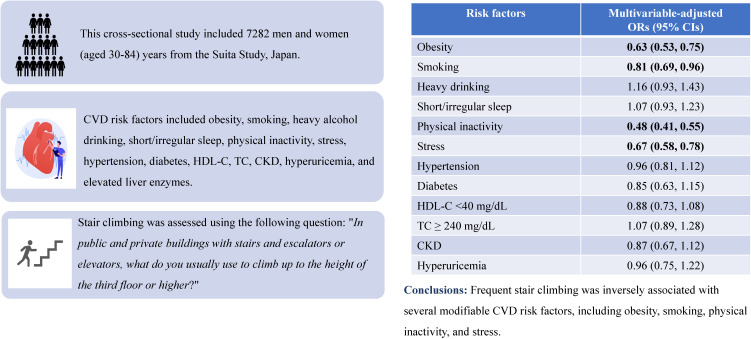

## Introduction

Cardiovascular disease (CVD) continues to stand as the primary contributor to morbidity and mortality both in Japan and worldwide [1]. Yet, the effective management of modifiable risk factors associated with CVD could play a pivotal role in alleviating its burden [2, 3].

Stair climbing is a readily available form of physical activity with potential cardiovascular benefits [4]. A previous study showed that a 5-week stair climbing training was associated with a 9.4% increase in aerobic capacity, an indicator of cardiorespiratory fitness, but no significant changes were noticed in blood pressure, lipid concentrations, or body composition [5]. Stair climbing training for 8 weeks displayed a 17.1% increase in aerobic capacity and a 7.7% reduction in low-density lipoprotein cholesterol (LDL-C), yet did not improve other lipid profile items [6]. A 12-week stair climbing intervention led to an increase in aerobic capacity by 9.2% and decreases in waist circumference, body weight, fat mass, diastolic blood pressure, and LDL-C by 1.7%, 0.7%, 1.5%, 1.8%, and 3.0%, respectively, however, the effect of stair climbing faded after six months [7]. A cross-sectional study demonstrated that refraining from stair climbing was associated with a 90% elevation in metabolic syndrome odds among late middle-aged men and women from the UK [8]. In a previous prospective cohort study, the highest frequency of stair climbing was associated with a 31% decline in atrial fibrillation risk among Japanese people [9]. Among older adults from Japan and the US, stair climbing was inversely associated with all-cause and cancer mortality, while the association with CVD mortality was inconsistent [10, 11]. Two recent studies have reported a dose-response reduction in the incidence of CVD among stair climbers in Japan and the UK; yet, this association was attenuated after adjustment for lifestyle and medical conditions [12, 13], suggesting that the potential cardioprotective effect of stair climbing could be partly explained by its role in controlling CVD risk factors.

Of note, the definition of stair climbing varied across studies. The Suita Study defined it as climbing stairs to the third floor or higher in public and private buildings with stairs and escalators or elevators [9, 10, 12]. The Harvard Alumni Health Study defined it as the weekly number of floors climbed (one floor = two flights of stairs) [11]. The UK Biobank defined it as the daily frequency of climbing a flight of stairs (10 steps) at home [13].

Given that 1) modifying CVD risk factors is essential for CVD prevention [2, 3], 2) stair climbing is a sort of physical activity with potential health merits [4–8], 3) studies investigating the association between stair climbing and modifiable CVD risk factors are scarce with inconsistent results [4–8], and 4) relevant findings from Asian populations are lacking, we performed this study to investigate the association between stair climbing and several modifiable CVD risk factors in a Japanese sample.

## Methods

### Study design and population

In this cross-sectional study, we utilized baseline information obtained from the Suita Study. As described elsewhere [14, 15], this dataset was compiled from a representative sample of urban Japanese residents situated in Suita City, Osaka. The National Cerebral and Cardiovascular Center (NCVC) oversaw the examination of two distinct cohorts, chosen at random based on sex and a 10-year age bracket. These examinations took place in 1989 (n = 6485) and 1996 (n = 1329), along with an additional group of volunteers recruited between 1992 and 1998 (n = 546). Respondents participated by completing a questionnaire assessing lifestyle and medical history and undergoing comprehensive medical assessments, including the collection of blood and urine samples. From a total of 8360 participants, we excluded individuals who did not partake in the assessment, had a documented history of stroke or myocardial infarction, or lacked data about stair climbing. Ultimately, this left us with 7282 participants, aged 30–84 years, who were included in the subsequent analysis.

### Assessment of cardiovascular disease risk factors

After reviewing previous literature [16, 17], the following CVD risk factors were examined: obesity, smoking, heavy alcohol drinking, short/irregular sleep, physical inactivity, stress, hypertension, diabetes, decreased high-density lipoprotein cholesterol (HDL-C), increased total cholesterol (TC), chronic kidney diseases (CKD), hyperuricemia, and elevated liver enzymes. Smoking, alcohol drinking, sleep, physical activity, and stress were self-reported using an interview questionnaire. Physical activity was assessed using a simple question: *“Do you practice physical activity?”* with *“yes”* and *“no”* as responses. Stress was also assessed using the following question: *“Is your daily life stressful?”* with *“yes”*, *“no”*, and *“not sure”* as responses. Obesity and hypertension were assessed by clinical examinations, while diabetes, HDL-C, TC, CKD, hyperuricemia, and liver enzymes were assessed using blood examinations. The medical profiles of all participants were reviewed to determine medical conditions that might have been undetected during the health check-up.

The following definitions for CVD risk factors were applied: obesity: body mass index (BMI) ≥ 25 kg/m^2^; smoking: current smoking (any quantity); heavy alcohol consumption: drinking alcohol ≥ 2 gou/day for men or ≥ 1.5 gou/day for women (1 gou = 23 g alcohol); short sleep: sleep ≤ 6 hours/night; hypertension: blood pressure ≥ 140/90 mmHg or medications; diabetes: fasting blood glucose ≥ 126 mg/dL or medications; decreased HDL-C: HDL-C < 40 mg/dL; increased TC: TC ≥ 240 mg/dL; CKD: estimated glomerular filtration rate (eGFR) < 60 ml/min/1.73 m^2^; hyperuricemia: uric acid > 7 mg/dL; and abnormal liver enzymes: glutamic-pyruvic transaminase (GPT) > 50 U/L, glutamic-oxaloacetic transaminase (GOT) > 50 U/L, or gamma-glutamyl transferase (GGT) > 100 U/L.

### Assessment of stair climbing

The following question in the baseline questionnaire was used to assess stair climbing: *“In public and private buildings with stairs and escalators or elevators, what do you usually use to climb up to the height of the third floor or higher?”* The responses were *“I use stairs most of the time (Stairs ≥ 80%)”*, *“I use stairs more than escalators or elevators (Stairs 60–79%)”*, *“half-half (Stairs 40–59%)”*, *“I use escalators or elevators more than stairs (Stairs 20–39%)”*, and *“I use escalators or elevators most of the time (Stairs < 20%)”*. The same question was used to assess stair climbing in previous studies [9, 10, 12].

### Statistical analysis

Due to the relatively limited number of participants in the stair-climbing groups 60–79% and ≥80%, we merged them into one group (≥60%). Logistic regression was used to calculate the odds ratios (ORs) and their 95% confidence intervals (95% CIs) of CVD risk factors in participants who reported stair climbing 20–39%, 40–59%, and ≥60% compared to <20% of the time. Two regression models were applied: model I adjusted for age and sex and model II further adjusted for obesity, smoking, alcohol consumption, sleep duration, physical activity, stress, hypertension, diabetes, HDL-C, TC, CKD, hyperuricemia, and liver enzymes. In a separate analysis, ORs (95% CIs) were calculated for stair climbing ≥20% of the time (20–39%, 40–59%, and ≥60% combined) compared to <20% of the time. To examine the potential impact of physical activity and age group on the association between stair climbing and CVD risk factors, we stratified the results and calculated the corresponding p-values for interaction. The interaction analysis was adjusted for variables in model II. SAS version 9.4 software (SAS Institute Inc, Cary, NC) was used for statistical analyses.

## Results

In the age- and sex-adjusted regression models, stair climbing was negatively associated with obesity (BMI ≥ 25 kg/m^2^), smoking, physical inactivity, stress, and decreased HDL-C: ORs (95% CIs) for the highest (>60% of the time) versus the lowest (<20% of the time) stair climbing frequencies were 0.62 (0.53, 0.74), 0.80 (0.68, 0.94), 0.47 (0.41, 0.54), 0.69 (0.60, 0.79), and 0.76 (0.63, 0.92), respectively (p-trends < 0.05). After further adjustment for lifestyle and clinical factors, the associations remained statistically significant for obesity, smoking, physical inactivity, and stress: ORs (95% CIs) = 0.63 (0.53, 0.75), 0.81 (0.69, 0.96), 0.48 (0.41, 0.55), and 0.67 (0.58, 0.78), respectively (p-trends < 0.05), but were attenuated for decreased HDL-C: OR (95% CI) = 0.88 (0.72, 1.07) (p-trend = 0.216). On the other hand, stair climbing was not associated, in both regression models, with heavy alcohol consumption, short sleep duration or irregular sleep, hypertension, diabetes, elevated TC, CKD, hyperuricemia, and elevated liver enzymes (Table 1).

**Table 1:** The association between stair climbing and modifiable cardiovascular disease risk factors (n = 7282)

**Risk factors**		**Stair climbing**				

		**<20%**	**20–39%**	**40–59%**	**≥60%**	**P-trend**
Number of participants		1773	1787	1967	1755	--
Age (mean ± Sd), years		56.7 ± 14.1	55.0 ± 13.4	55.1 ± 12.4	54.5 ± 12.2	<0.001
Men, %		41.0	42.4	45.0	57.3	<0.001
Obesity	%	23.8	20.4	19.0	16.7	--
	Model I	1 (Reference)	0.82 (0.70, 0.96)	0.75 (0.64, 0.88)	0.62 (0.53, 0.74)	<0.001
	Model II	1 (Reference)	0.82 (0.69, 0.97)	0.73 (0.62, 0.87)	0.63 (0.53, 0.75)	<0.001
Smoking	%	29.7	26.5	26.8	33.1	--
	Model I	1 (Reference)	0.77 (0.65, 0.91)	0.74 (0.63, 0.87)	0.80 (0.68, 0.94)	0.006
	Model II	1 (Reference)	0.76 (0.64, 0.90)	0.75 (0.63, 0.88)	0.81 (0.69, 0.96)	0.015
Heavy drinking	%	12.6	13.7	14.8	18.5	--
	Model I	1 (Reference)	1.05 (0.85, 1.30)	1.11 (0.91, 1.37)	1.12 (0.92, 1.37)	0.217
	Model II	1 (Reference)	1.13 (0.90, 1.41)	1.18 (0.95, 1.46)	1.16 (0.93, 1.43)	0.154
Short/irregular sleep	%	37.1	36.6	35.7	35.4	--
	Model I	1 (Reference)	1.02 (0.89, 1.16)	0.98 (0.86, 1.12)	1.03 (0.90, 1.19)	0.807
	Model II	1 (Reference)	1.03 (0.89, 1.18)	1.00 (0.87, 1.15)	1.07 (0.93, 1.23)	0.472
Physical inactivity	%	68.7	63.2	56.8	50.0	--
	Model I	1 (Reference)	0.77 (0.67, 0.89)	0.59 (0.52, 0.68)	0.47 (0.41, 0.54)	<0.001
	Model II	1 (Reference)	0.79 (0.68, 0.91)	0.61 (0.53, 0.69)	0.48 (0.41, 0.55)	<0.001
Stress	%	45.2	42.5	42.5	38.3	--
	Model I	1 (Reference)	0.83 (0.72, 0.95)	0.80 (0.70, 0.92)	0.69 (0.60, 0.79)	<0.001
	Model II	1 (Reference)	0.82 (0.71, 0.94)	0.79 (0.69, 0.90)	0.67 (0.58, 0.78)	<0.001
Hypertension	%	34.4	30.7	33.6	28.9	--
	Model I	1 (Reference)	0.93 (0.80, 1.08)	1.08 (0.94, 1.26)	0.86 (0.74, 1.01)	0.254
	Model II	1 (Reference)	0.96 (0.82, 1.12)	1.17 (1.01, 1.36)	0.96 (0.81, 1.12)	0.801
Diabetes	%	6.3	5.2	5.0	5.0	--
	Model I	1 (Reference)	0.85 (0.64, 1.13)	0.83 (0.62, 1.10)	0.79 (0.59, 1.06)	0.121
	Model II	1 (Reference)	0.87 (0.65, 1.17)	0.84 (0.63, 1.12)	0.85 (0.63, 1.15)	0.274
HDL-C < 40 mg/dL	%	15.6	14.2	13.9	14.3	--
	Model I	1 (Reference)	0.90 (0.74, 1.07)	0.84 (0.70, 1.01)	0.76 (0.63, 0.92)	0.004
	Model II	1 (Reference)	0.97 (0.80, 1.18)	0.96 (0.79, 1.16)	0.88 (0.73, 1.08)	0.226
TC ≥ 240 mg/dL	%	18.8	18.3	19.4	16.7	--
	Model I	1 (Reference)	1.03 (0.87, 1.22)	1.14 (0.96, 1.34)	1.05 (0.88, 1.25)	0.411
	Model II	1 (Reference)	1.04 (0.87, 1.23)	1.15 (0.97, 1.36)	1.07 (0.89, 1.28)	0.302
CKD	%	9.7	8.9	7.6	7.0	--
	Model I	1 (Reference)	1.00 (0.80, 1.26)	0.86 (0.68, 1.09)	0.85 (0.66, 1.09)	0.111
	Model II	1 (Reference)	1.00 (0.79, 1.26)	0.89 (0.70, 1.14)	0.87 (0.67, 1.12)	0.191
Hyperuricemia	%	8.9	9.3	8.3	10.9	--
	Model I	1 (Reference)	1.02 (0.80, 1.29)	0.84 (0.66, 1.07)	0.91 (0.72, 1.15)	0.206
	Model II	1 (Reference)	1.03 (0.80, 1.32)	0.82 (0.64, 1.05)	0.96 (0.75, 1.22)	0.364
Elevated liver enzymes	%	9.3	8.7	9.3	10.8	--
	Model I	1 (Reference)	0.91 (0.72, 1.15)	0.93 (0.74, 1.17)	0.93 (0.74, 1.17)	0.620
	Model II	1 (Reference)	0.94 (0.74, 1.21)	0.97 (0.76, 1.23)	1.03 (0.81, 1.31)	0.754

Model I: Odds ratios (95% confidence intervals) were adjusted for age and sex

Model II: Odds ratios (95% confidence intervals) were adjusted for age, sex, obesity, smoking, drinking, sleep, physical activity, stress, hypertension, diabetes, HDL-C, TC, CKD, hyperuricemia, and liver enzymes

CKD: Chronic kidney disease; HDL-C: High-density lipoprotein-cholesterol; TC: Total cholesterol

When we combined stair climbing frequencies 20–39%, 40–59%, and ≥60% of the time into one group (≥20% of the time) and compared it to stair climbing <20% of the time, the results did not materially change. When the results were stratified by the status of physical activity, we did not detect superior preventive effects amongst participants who reported physical activity. The multivariable-adjusted ORs (95% CIs) displaying the associations of stair climbing ≥20% of the time with obesity, smoking, stress, and decreased HDL-C were 0.73 (0.64, 0.83), 0.77 (0.67, 0.88), 0.76 (0.68, 0.85), and 0.94 (0.80, 1.10) in all participants, 0.71 (0.60, 0.84), 0.68 (0.54, 0.86), 0.72 (0.63, 0.83), and 0.98 (0.81, 1.19) in physically-inactive participants, and 0.77 (0.61, 0.98), 0.81 (0.68, 0.96), 0.83 (0.68, 1.00), and 0.89 (0.68, 1.17) in physically-active participants, respectively (p-interactions > 0.10). The results remained consistent across age groups. The multivariable-adjusted ORs (95% CIs) showing the associations of stair climbing ≥20% of the time with obesity, smoking, physical inactivity, and stress among participants <60 years were 0.75 (0.63, 0.90), 0.75 (0.63, 0.90), 0.62 (0.53, 0.73), and 0.78 (0.67, 0.91), respectively, and among participants ≥60 years were 0.78 (0.63, 0.97), 0.78 (0.63, 0.97), 0.61 (0.51, 0.73), and 0.72 (0.60, 0.87), respectively (p-interactions > 0.10).

## Discussion

Understanding how daily activities like stair climbing can impact CVD risk factors may inform public health campaigns and individual health promotion strategies and encourage preventive medicine efforts. Our results endorsed the growing evidence indicating potential cardiovascular benefits for stair climbing. Frequent stair climbing was associated with lower odds of obesity, smoking, physical inactivity, and stress among Japanese people. These associations remained statistically significant among older adults.

Stair climbing is a sort of physical activity [4], and previous reports showed negative associations between physical activity and the risk of obesity [18], smoking [19], and stress [20]. Thus, it could be speculated that those who reported higher frequencies of stair climbing had lower odds of obesity, smoking, and stress because they were already physically active. However, when we stratified our results by the status of physical activity, no superior preventive effects were detected amongst participants who reported physical activity, suggesting that the association between stair climbing and CVD risk factors was independent of practicing physical activity.

However, there is a possibility that participants who used stairs more frequently had more concern about health, better health awareness, or more interest in healthy behaviors. Health perceptions are associated with health-promoting behaviors [21]. Besides, it could be suggested that respondents might have perceived the stair climbing question as a tool assessing their intention to use stairs or attitude toward stair climbing rather than actual behavior. Still, a meta-analysis of 13 randomized clinical trials showed that the application of the implementation intentions strategy to promote physical activity was capable of increasing physical activity practice [22]. Moreover, a positive attitude towards physical activity was shown in previous studies to be inversely associated with obesity [23] and smoking [24].

The primary strengths of this study are the recruitment of a study population that accurately represents urban Japanese individuals, the application of standardized techniques to define most CVD risk factors, and the control for numerous confounding factors. However, many limitations warrant consideration. First, due to the cross-sectional design and the absence of temporality, it is obscure for our results to imply causality. Second, the reliance on self-reported data for stair climbing practice introduces the potential for recall bias. Third, the questionnaire specifically inquired whether participants were climbing stairs to the third floor or higher, potentially leading to an assignment of individuals who only climbed to the first or second floors into the <20% group. This classification approach could have introduced differential misclassification and weakened the observed cardioprotective impact of stair climbing. Fourth, variations in floor heights and the number of stairs per floor across different buildings were not taken into account. Fifth, physical activity and stress were assessed using simple questions rather than validated scales. Sixth, the absence of data concerning musculoskeletal and neurological disorders that could potentially limit mobility represents another limitation. However, our analysis showed that age and physical activity, which are strongly correlated with physical mobility, did not impact the association between stair climbing and CVD risk factors, suggesting that such disorders had no determining impact on the findings.

In conclusion, frequent stair climbing was inversely associated with several modifiable CVD risk factors, including obesity, smoking, physical inactivity, and stress. These associations were irrespective of age and the status of physical activity. Our findings can inform the development of risk-reduction strategies tailored to populations with the above-mentioned CVD risk factors. Still, future prospective cohort studies should investigate the mediating and moderating effects of CVD risk factors on the association between stair climbing and CVD.

## Declarations

### Availability of data and materials

Data could not be provided for ethical reasons.
